# Inverse association of vitamin D_3_ levels with lung cancer mediated by genetic variation

**DOI:** 10.1002/cam4.1444

**Published:** 2018-05-03

**Authors:** Majda Haznadar, Kristopher W. Krausz, Ezra Margono, Christopher M. Diehl, Elise D. Bowman, Soumen Kanti Manna, Ana I. Robles, Bríd M. Ryan, Frank J. Gonzalez, Curtis C. Harris

**Affiliations:** ^1^ Laboratory of Human Carcinogenesis Center for Cancer Research National Cancer Institute Bethesda Maryland 20892; ^2^ Laboratory of Metabolism Center for Cancer Research National Cancer Institute Bethesda Maryland 20892; ^3^ Biophysics and Structural Genomics Division Saha Institute of Nuclear Physics (HBNI) Kolkata 700064 India

**Keywords:** 1,25(OH)D, 25(OH)_2_D, CYP24A1, lung cancer status, serum, SNP, vitamin D

## Abstract

Vitamin D is an essential micronutrient required for normal physiological function and recognized for its role regulating calcium metabolism. Recent work is beginning to emerge demonstrating a role for vitamin D in chronic illnesses, such as cancer. Circulating serum levels of 25(OH)D_2/3_ were quantitatively measured using sensitive ultraperformance liquid chromatography coupled to tandem mass spectrometry (UPLC‐MS/MS) in 406 lung cancer cases and 437 population controls, while 1,25(OH)_2_D_2/3_ levels were measured in a subset of 90 cases and 104 controls using the same method, from the NCI‐MD case–control cohort. 25(OH)D_3_ levels were inversely associated with lung cancer status across quartiles (Q2 vs. Q1: OR_adjusted_ = 0.5, 95% CI = 0.3–0.8; Q3 vs. Q1: OR_adjusted_ = 0.5, 95% CI = 0.3–0.8; Q4 vs. Q1: OR_adjusted_ = 0.5, 95% CI = 0.2–0.9; *P*_trend_ = 0.004). Levels of 1,25(OH)_2_D_3_ were also inversely associated with lung cancer status (Q2 vs. Q1: OR_adjusted_ = 0.2, 95% CI = 0.03–0.7; Q3 vs. Q1: OR_adjusted_ = 0.1, 95% CI = 0.01–0.4; Q4 vs. Q1: OR_adjusted_ = 0.04, 95% CI = 0.01–0.3; *P*_trend_<0.0001). Although the observed trends were similar for the 25(OH)D_2_ (*P*_trend_ = 0.08), no significant associations were seen between vitamin D_2_ and lung cancer status. Additionally, genotyping of 296 SNPs in the same subjects resulted in findings that 27 SNPs, predominantly in CYP24A1 and VDR genes, were significantly associated with lung cancer status, affected mRNA expression, and modulated vitamin D levels. These findings suggest a protective role for vitamin D_3_ in lung cancer, with similar trends but insignificant findings for D_2_. Vitamin D_3_ levels appeared to be modulated by genetic variation in CYP24A1 and VDR genes. Additional research to illuminate the mechanism(s) through which vitamin D exacerbates effects against lung carcinogenesis is warranted.

## Introduction

Lung cancer is the leading cause of cancer deaths in men and women in the United States 1. While five‐year survival is 54.0% in localized cases, it drops to 26.5% for regional and 4.0% for distant dissemination at diagnosis 2. Novel mechanisms of lung carcinogenesis are emerging, including research demonstrating a role for vitamin D in lung cancer 3. Vitamin D is an essential micronutrient required for normal physiological function. The majority of the known effects of vitamin D are related to skeletal health outcomes, but the relatively recent Institute of Medicine (IOM) report has highlighted the need for more research to explore the role of vitamin D in cancer and other outcomes 4. Vitamin D exists in two forms, vitamin D_2_ (ergocalciferol), a plant derivative often found in supplements, and vitamin D_3_ (cholecalciferol), produced in the skin upon exposure to UV light 5. Both forms of vitamin D are metabolized to inactive 25‐hydroxyvitamin D (25(OH)D) in the liver and converted to its hormonally active form, 1,25(OH)_2_D in the kidneys. The active 1,25(OH)_2_D increases calcium absorption and exerts its effects by interacting with the vitamin D receptor–retinoic acid X receptor complex (VDR‐RXR) to control genes responsible for cell proliferation, differentiation, apoptosis, and angiogenesis 6. Vitamin D_3_ is metabolized with a higher efficiency compared with D_2_. There are several biological mechanisms that contribute to the superior absorbability and efficacy of vitamin D_3_: in the liver, inactive D_3_ is more readily metabolized into a bioactive form of vitamin D that is easily converted to its hormone form in the kidneys. It takes a significantly longer time to make this hepatic conversion with vitamin D_2_ 7.

Significant evidence exists to support an antiproliferative role for vitamin D in the context of cancer. In lung cancer specifically, 1,25(OH)_2_D_3_ was shown to inhibit cell proliferation in cell lines 8. Additionally, treatment with 1,25(OH)_2_D_3_ decreased cancer growth in the tobacco‐specific carcinogen, nitrosamine, 4‐(methylnitrosamino)‐1‐(3‐pyridyl)‐1‐butanone (NKK)‐induced lung tumors in mice 9, and reduced metastasis in VDR knockout mice compared to the wild‐type controls in the Lewis lung carcinoma (LLC) model 10.

Vitamin D metabolism may also be affected by genetic factors. There have been several studies published that have demonstrated that single nucleotide polymorphisms (SNPs) in genes involved in vitamin D metabolism are associated with lung cancer 11, 12. Notably, significant associations with lung cancer have been observed with variants in vitamin D receptor (VDR) and cytochrome P450 CYP24A1 genes 13. Furthermore, CYP24A1 has been shown to be overexpressed in lung, as well as other cancer types, and was found to be an independent prognostic marker of survival in patients with lung adenocarcinoma 14. The aforementioned observations point to a crucial role of VDR and CYP24A1 in vitamin D metabolism, possibly through modulating vitamin D levels, which may explain their observed associations with lung cancer.

In 2011, the Institute of Medicine (IOM) updated the Dietary Reference Intakes (DRIs) for vitamin D. Based on bone health, a recommended dietary allowance (RDA) is 600 IU (15 *μ*g/day) for adults below 70 years of age, and 800 IU (20 *μ*g/day) for those above 70 years, in order to achieve optimal serum 25(OH)D levels of 50 nmol/L (20 ng/mL) 4. Throughout the report, D_2_ and D_3_ forms were frequently discussed interchangeably, as the distinction in many reviewed studies was missing. Bone health was the only health outcome with evidence to provide support for DRI development. Other health outcomes, including those related to cancer, could not be reliably linked with vitamin D intake, highlighting the need for more research to explore the role of vitamin D in nonskeletal health outcomes.

A number of studies investigated the relationship between serum 25(OH)D levels and cancer status and risk, with contradictory evidence. The most compelling data for inverse associations between circulating total 25(OH)D and cancer risk were shown in colorectal and breast cancers. Higher levels of circulating total 25(OH)D were associated with reduced risk in breast 15, 16 and colorectal cancer 16, 17, 18, 19, whereas no significant associations were observed for prostate cancer risk 20, 21, 22. The evidence for association between 25(OH)D levels and lung cancer is similarly inconsistent, with studies reporting both protective 23, 24, 25, and null associations 26, 27. Significantly, some of the heterogeneity across studies could be related to methodological limitations. Firstly, studies rarely distinguish between 25(OH)D and 1,25(OH)_2_D metabolites, such that the measurement reported is largely total vitamin D. Secondly, 1,25(OH)_2_D is present in the low pg/mL range in the circulation, making quantitative analysis challenging using traditional methods. Thirdly, the majority of the population‐based studies reporting the relationship between vitamin D and cancer have been conducted using radioimmunoassays (RIAs) and chemiluminescence immunoassays (CLIAs), which do not have the ability to distinguish between the D_2_ and D_3_ forms and suffer from endogenous and exogenous interferents, such as autoimmune rheumatoid factor (RF), human anti‐mouse antibodies (HAMA), and many drugs. Recently, concerns regarding the inconsistencies in vitamin D measurements across studies due to insufficiently sensitive and nonspecific methods, as well as not using internationally recognized reference standards that would allow for cross‐laboratory comparisons, have been raised by the U.S. Preventive Services Task Force (USPSTF) 28. In this study, we leveraged sensitive ultraperformance liquid chromatography coupled to tandem mass spectrometry (UPLC‐MS/MS) to quantitatively measure 25(OH)D_2/3_ (406 non‐small‐cell lung cancer (NSCLC) cases and 437 controls) and 1,25(OH)_2_D_2/3_ in a representative subset comprising 90 cases and 104 controls in a case–control study design. We further genotyped 296 SNPs in the same subjects (*N* = 761) in CYP2R1, CYP27A1, CYP27B1, CYP24A1, and VDR genes directly involved in vitamin D metabolism, with an objective to elucidate variants that may modulate vitamin D levels, thereby potentially explaining their associations with lung cancer. This is the first study to date to examine all metabolites of vitamin D using an ultrasensitive method and to conduct comprehensive genotyping of variants in the vitamin D metabolism network in the same subjects. The aforementioned allowed for an investigation of the association between this important prohormone and lung cancer with a higher accuracy, while also illuminating important biology at a genetic level.

## Methods

### Study subjects

The study population and eligibility criteria were previously described 29, 30. Patients with non‐small‐cell lung cancer (NSCLC) (*n* = 406) were recruited from hospitals in the Baltimore, MD, metropolitan area between 1998 and 2007. All lung cancer diagnoses were histologically confirmed NSCLC cases. Population controls (*n* = 437) were recruited from the same area by screening lists from the Department of Motor Vehicles (DMVs) to match cases by age and gender. Blood was separated into blood components (serum, plasma, buffy coat, and red blood cells) at interview, and frozen at the University of Maryland before being sent to the Laboratory of Human Carcinogenesis (LHC) for storage until use. This study was approved by the Institutional Review Boards of the National Cancer Institute, the Johns Hopkins University School of Medicine, University of Maryland Baltimore, MedStar Research Institute, the Research Ethics Committee of Bon Secours Baltimore Health System, and Sinai Hospital.

Quantitation of 25(OH)D_2/3_ was conducted in 843 samples (406 cases and 437 population controls). Measurement of 1,25(OH)_2_D_2/3_ was conducted in a representative subset of 194 samples with a similar distribution of the key variables when compared to the larger set. The study designs of the overall cohort and the subset are depicted in Table**** 1 and Table S1, respectively.

**Table 1:** Demographic and clinical characteristics of the NCI‐MD case–control set

Characteristics	Cases	Controls	Total	*P‐*value^b^
*n*	406	437	843	0.30
Age, mean ± SE	66.3 ± 10.0	67.0 ± 8.9	66.6 ± 9.4	
Race, *n* (%)				
African American	100 (25)	193 (44)	293 (35)	<0.0001
European American	306 (75)	244 (56)	550 (65)	
Gender, *n* (%)				
Male	214 (53)	234 (54)	295 (56)	0.81
Female	192 (47)	203 (44)	229 (44)	
Smoking status, *n* (%)				
Current	191 (47)	52 (12)	243 (29)	<0.0001
Former	186 (46)	209 (48)	395 (47)	
Never	29 (7)	176 (40)	205 (24)	
Pack‐years; mean ± SE	42.6 ± 1.4	15.2 ± 1.0	28.5 ± 1.0	<0.0001
Histology, *n* (%)				
Adenocarcinoma	202 (49)			
Squamous cell carcinoma	108 (27)			
Non‐small‐cell carcinoma	96 (24)			
Stage^a^, *n* (%)				
I	136 (34)			
II	56 (14)			
III	37 (9)			
IV	37 (9)			
Unknown	140 (34)			

a Only cases containing pathology reports were staged. Staging presented in the table is based on the 7th edition of the AJCC staging manual, wherein all cases with sufficient tumor size, metastases status, and lymph node involvement data previously staged based on the 6th edition were restaged to the 7th edition for consistency.

b Two‐sided chi‐square test (categorical) or *t*‐test (continuous).

### Sample preparation

Measurements of 25(OH)D_2/3_ were conducted in human serum samples using a protocol developed for the Waters UPLC‐MS/MS platform 31. Briefly, 15 *μ*L of 0.2 m ZnSO_4_ with 1 *μ*mol/L hexadeuterated 25(OH)D_3_ (Medical Isotopes, Waltham, MA) as an internal standard was added to 150 *μ*L of serum and vortexed for 10 sec. Three hundred microliters of methanol was added and vortexed for 10 sec to precipitate out the proteins, upon which 750 *μ*L of hexane was added to extract vitamin D metabolites. After 30 sec of vortexing, samples were centrifuged for 5 min at 14,000 × *g,* and the hexane layer was removed and placed in a total recovery vial (Waters, Milford, MA). Samples were evaporated to dryness using a SpeedVac Concentrator (Thermo Scientific) at room temperature (RT) and reconstituted with 75 *μ*L of 70% methanol in water. To carry out the quantitation in serum, standard curves were generated using 1 × 10^−5^, 5 × 10^−4^, 1 × 10^−3^, 0.01, 0.02, 0.05, 0.1, 0.2, 0.5, and 1.0 *μ*mol/L of 25(OH)D_2_ and 25(OH)D_3_ in 150 *μ*L of 4% BSA and processed as described above for serum samples (dynamic range of the 25(OH)D assay was from 0.01 nM to 1 *μ*mol/L). Furthermore, standard reference materials (SRM) 972a from the National Institute of Standards and Technology (NIST), containing a set of serum samples with certified reference values for 25(OH)D_2_ and 25(OH)D_3_ 32, were processed as described above to standardize measurements of 25(OH)D metabolites in order to allow for cross‐laboratory comparisons. Therefore, 25(OH)D_2/3_ measurements are traceable to NIST SRM 972a. There are no internationally recognized standards for 1,25(OH)_2_D_2_ or 1,25(OH)_2_D_3_.

Quantitation of 1,25(OH)_2_D_2/3_ in serum samples was carried out using 1,25(OH)_2_D ImmunoTube^®^ Extraction Kit (Immundiagnostik AG, Bensheim, Germany). Extraction was performed per manufacturer's protocol. Briefly, 10 *μ*L of 0.03 *μ*mol/L hexadeuterated 1,25(OH)_2_D_3_ (Medical Isotopes Inc., Pelham, NH) internal standard was added to 500 *μ*L of serum and transferred to ImmunoTubes^®^. Serum samples were mixed by orbital rotation for 1 hr at RT and washed twice with 500 *μ*L of wash solution. Metabolites were eluted with 250 *μ*L of elution reagent and collected in 12 x 75 mm borosilicate glass test tubes (Kimble Chase, Vineland, NJ). Samples were evaporated using a SpeedVac and derivatized to improve the sensitivity for detection of 1,25(OH)_2_D_2/3_ with 50 *μ*L of 9 mmol/L 4‐phenyl‐1,2,4‐triazoline‐3,5‐dione (PTAD) (Sigma‐Aldrich, St. Louis, MO) in acetonitrile. After 30 min of incubation at RT, 50 *μ*L of UPLC‐grade water was added to quench unreacted PTAD. Next, samples were vortexed briefly, centrifuged at 14,000 × *g* at 4°C for 5 min, and then 5 *μ*L of sample was injected onto the mass spectrometer. For quantitation, standard curves were generated using 5 × 10^−5^, 1 × 10^−4^, 5 × 10^−3^, 0.01, 0.5, 1, 5, 10, 50, 100, and 500 nM of 1,25(OH)_2_D_2_ (Medical Isotopes Inc., Pelham, NH) and 1,25(OH)_2_D_3_ (Cayman Chemical, Ann Arbor, Michigan) in 500 *μ*L of 4% BSA and processed as described above for serum samples above (dynamic range of 1,25(OH)_2_D assay was from 0.05 pmol/L to 0.5 *μ*mol/L).

Of note, all 843 samples passed the quality control (QC) for the detection of the 25(OH)D_3_, while 295 (139 cases, 156 controls) samples had measurable 25(OH)D_2_. While a representative set of 194 samples was initially selected for quantitation of 1,25(OH)_2_D levels, a subset of those passed the QC: hexadeuterated 1,25(OH)_2_D_3_ internal control of a known concentration was spiked in all samples that were subsequently processed using ImmunoTube^®^ Extraction Kit. Samples that did not result in detectable levels of a hexadeuterated 1,25(OH)_2_D_3_ internal control were removed from the analysis, as endogenous 1,25(OH)_2_D levels could not be normalized to the internal control, and their levels could not be reliably determined. This may be in part due to defective or inefficient ImmunoTube^®^ extraction kits used for the isolation of 1,25(OH)_2_D. The aforementioned quality control assessment resulted in 116 (59 cases, 57 controls) with 1,25(OH)_2_D_3_ and 99 samples (51 cases, 48 controls) with 1,25(OH)_2_D_2_ measurements included in the analysis. Of note, demographic and clinical variables were not statistically different between the subjects whose 1,25(OH)_2_D level measurements passed the QC and those who did not. Therefore, the associations presented herein may be generalized to all 194 samples that were selected for profiling.

### Ultraperformance liquid chromatography coupled to tandem mass spectrometry

Quantitation of the metabolites was performed by multiple reaction monitoring (MRM) using a Waters Acquity^™^ UPLC system coupled to a Waters Xevo TQ triple quadrupole mass spectrometer (Waters Inc., Milford, MA) operated by MassLynx software. A Waters Acquity BEH C8 column (2.1 × 50 mm) was used for the quantitation of 25(OH)D_2/3_. UPLC was performed with the following (each buffer contained 2 mmol/L ammonium formate and 0.1% formic acid): The initial gradient contained 25% water and 75% methanol for 0.2 min, then a linear gradient to 5% water at 5 min, held until 5.5 min, and returned to initial conditions and held for 2 min for column equilibration. The flow rate was 0.4 mL/min, and the column temperature was maintained at 45°C. The instrument was operated in MRM electrospray ionization positive (ESI+) mode, with the following conditions: 2.5 kV capillary voltage, 150°C source temperature, desolvation gas flow rate of 850 L/h at 450°C, and cone gas flow of 50 L/hr. The total run time was 7 min. The following MRM transitions (m/z) were monitored as follows: 25(OH)D_3_ (401.3→105.0, 401.3→159.1, 401.3→257.0, 401.3→365.0, 401.3→383.3); 25(OH)D_2_ (413.4→83.0, 413.4→107.0, 413.4→337.0, 413.4→355.0, 413.4→395.0); hexadeuterated 25(OH)D_3_ (as internal standard) (407.3→105.0, 407.3→107.60, 407.3→159.1, 407.3→263.0, 407.3→371.0, 407.3→389.3). The optimal cone voltage and collision energy for each MRM transition were determined using IntelliStart software (Waters Inc., Milford, MA).

Separation of 1,25(OH)_2_D_2/3_ was performed as previously described 33. Briefly, a Waters Acquity BEH C18 column (2.1 × 50 mm) was used for metabolite separation. UPLC was performed with water and acetonitrile with 0.1% formic acid. Gradient, flow rate, ESI mode, and conditions were as described above. The column temperature was maintained at 40°C. The total run time was 5 min. The following MRM transitions (m/z) were monitored: 1,25(OH)_2_D_3_ (574.3→314.1, 592.3→314.1); 1,25(OH)_2_D_2_ (586.3→314.3.0); hexadeuterated 1,25(OH)_2_D_3_ (as internal standard) (580.3→314.1). The optimal cone voltage and collision energy for each MRM transition were determined using IntelliStart software.

All data were processed using TargetLynx software (Waters Inc., Milford, MA), to generate results from the acquired chromatographic data, permitting accurate quantitation, and review of results, including evaluation of data quality and analyte confirmation. Internal standard normalized areas under the peak (response) from authentic standard solutions were used to build calibration curves, which were then used for the quantitation of metabolites in serum samples.

### SNP genotyping

Genomic DNA was extracted from whole blood samples stored at −80°C using DNeasy Blood & Tissue Kit (Qiagen, Germantown, MD) and set at 50 ng/*μ*L.

Single nucleotide polymorphisms (SNPs), validated by sequencing in the 1000 Genomes project, with a minor allele frequency of ≥5% in enzymes directly involved in vitamin D metabolism (CYP2R1, CYP27A1, CYP27B1, CYP24A1, and VDR) were selected for genotyping. Of 365 SNPs that passed the aforementioned criteria, 296 SNPs passed the primer design quality control, for which SNPType assays were obtained from Fluidigm (Fluidigm Corp., South San Francisco, CA). Genotyping was performed on a 96.96 Dynamic Array IFC chip (Fluidigm, PN BMK‐M‐96.96GT) using a BioMark instrument (Fluidigm Corp.) per manufacture's specifications and protocol.

The data were analyzed using the Fluidigm Genotyping Analysis Software with Auto‐Call Analysis feature and the NTC Data Normalization Method with a Confidence Threshold set at the default of 65. There were 761 samples that were genotyped across 296 unique SNPs. Fifteen percent of the replicated samples resulted in 99% intra‐ and 97% interplate reproducibility.

### Statistical analysis

All analyses were performed using Stata software, version 13 (Stata Statistical Software Release 13.1, College Station, TX). All reported *P* values were two‐sided, and all *P* values less than or equal to 0.05 were considered statistically significant. Univariable comparisons between cases and controls were performed using the chi‐square test on categorical variables and using the Student's *t*‐test (normally distributed data) or Kruskal–Wallis test (non‐normally distributed data) on continuous variables. Mann–Whitney (two‐sample Wilcoxon rank sum) test was used to analyze differences of 25(OH)D_2/3_ and 1,25(OH)_2_D_2/3_ levels between cases and controls. Student's *t*‐test was used for CYP24A1 expression analysis between tumor and adjacent nontumor tissue. One‐way ANOVA was used to analyze CYP24A1 expression and 25(OH)D_3_ levels across rs10623012 genotypes.

Unconditional logistic regression models were used to estimate odds ratios (ORs) and 95% confidence intervals (CIs) for the association of lung cancer with 25(OH)D_2/3_ and 1,25(OH)_2_D_2/3_, categorized by quartile values based on the control subjects, 25th, 50th, and 75th percentile quartiles: 25(OH)D_3,_ 10.4, 15.5, and 25.1 ng/mL; 25(OH)D_2_, 23.8, 27.2, and 32.3 ng/mL; 1,25(OH)_2_D_3_, 2.2, 5.5, and 12.9 pg/mL; 1,25(OH)_2_D_2_, 1.9, 4.9, and 13.7 pg/mL. Levels of 25(OH)D_3_ were also categorized to high and low based on the IOM‐reported reference value of <20 ng/mL indicating vitamin D deficiency. Multivariable models of vitamin D levels were adjusted for age at diagnosis, gender (male/female), race (European/African Americans), cigarette smoking status (never/former/current), amount smoked among smokers (pack‐years), interview year, blood collection month, and self‐reported use of vitamin D supplements (yes/no). Subgroup analyses were performed by stratifying on race (European Americans and African Americans). Multivariable models of SNPs were adjusted for age, gender, and race.

Smokers were categorized as never, former, and current. A never smoker was defined as a person who smoked less than 100 cigarettes in his lifetime, a former smoker was defined as a person who had quit smoking more than 1 year before the interview, and a current smoker was defined as a person who reported smoking within the year prior to the interview, or before. Regarding the self‐reported use of vitamin D supplements, this variable was dichotomized into those who self‐reported taking vitamin D or multivitamins containing vitamin D (“yes”), and those who self‐reported *not* taking the aforementioned (“no”).

## Results

### Serum vitamin D levels

First, we evaluated the difference between 25(OH)D_2/3_ and 1,25(OH)_2_D_2/3_ levels among cases and controls, and found that levels are significantly lower in lung cancer cases when compared to the population controls for 25(OH)D_3_ and 1,25(OH)_2_D_3_ levels (*P* < 0.0001). Differences in vitamin D_2_ levels between the cases and the controls were less pronounced compared with vitamin D_3._ Levels of 25(OH)D_2_ were significantly lower in lung cancer cases (*P* = 0.05), whereas, although lower, 1,25(OH)_2_D_2_ levels were not significantly different between cases and controls (Table S2).

### Associations of vitamin D serum levels and lung cancer status

In multivariable unconditional logistic regression analyses, increasing levels of 25(OH)D_3_ were inversely associated with lung cancer status across quartiles when compared to the lowest quartile (Q2 vs. Q1: OR_adjusted_ = 0.5, 95% CI = 0.3–0.8, *P* = 0.002; Q3 vs. Q1: OR_adjusted_ = 0.5, 95% CI = 0.3–0.8, *P* = 0.003; Q4 vs. Q1: OR_adjusted_ = 0.5, 95% CI = 0.2–0.9, *P* = 0.02; *P*_trend_ = 0.004), indicating that high levels of vitamin D_3_ are protective in lung cancer (Table**** 2A).

**Table 2:** Logistic regression analysis of (A) 25(OH)D_3_ and (B) 1,25(OH)_2_D_3_ levels across quartiles

	Univariable				Multivariable			
	*N* (%) cases	*N* (%) controls	OR (95% CI)	*P*‐value^a^	*N* (%) cases	*N* (%) controls	OR (95% CI)^b^	*P*‐value^a^
(A) 25(OH)D_3_								
Q1_referent_	187 (46)	110 (25)	1.0		187 (46)	109 (25)	1.0	
Q2	94 (23)	104 (24)	**0.5 (0.4–0.8)**	**0.001**	91 (23)	104 (24)	**0.5 (0.3–0.8)**	**0.002**
Q3	73 (18)	113 (26)	**0.4 (0.3–0.6)**	**<0.0001**	73 (18)	113 (26)	**0.5 (0.3–0.8)**	**0.003**
Q4	52 (13)	110 (25)	**0.3 (0.2–0.4)**	**<0.0001**	51 (13)	107 (25)	**0.5 (0.2–0.9)**	**0.02**
		***P***_**trend**_ **<0.0001**						***P***_**trend**_ = **0.004**
(B) 1,25(OH)_2_D_3_								
Q1_referent_	41 (69)	14 (25)	1.0		41 (70)	14 (25)	1.0	
Q2	10 (17)	15 (26)	**0.2 (0.1–0.6)**	**0.004**	10 (17)	15 (27)	**0.2 (0.03–0.7)**	**0.01**
Q3	5 (9)	13 (23)	**0.1 (0.04–0.4)**	**0.001**	5 (9)	13 (23)	**0.1 (0.01–0.4)**	**0.004**
Q4	3 (5)	15 (26)	**0.1 (0.02–0.3)**	**<0.0001**	3 (5)	14 (25)	**0.04 (0.01–0.3)**	**0.001**
			***P***_**trend**_ **<0.0001**					***P***_**trend**_ **<0.0001**

CI, confidence interval; OR, odds ratio.

a **Statistically significant; ***P***‐value <0.05.**

b Multivariable unconditional logistic regression adjusted for age, gender, race, interview year, smoking status, pack‐years, blood collection month, and vitamin D supplement use.

As previously mentioned, levels of 1,25(OH)_2_D_3_ were measured in a subset of 90 cases and 104 controls. Increasing levels of 1,25(OH)_2_D_3_ were inversely and significantly associated with lung cancer status across all quartiles (Q2 vs. Q1: OR_adjusted_ = 0.2, 95% CI = 0.03–0.7, *P* = 0.01; Q3 vs. Q1: OR_adjusted_ = 0.1, 95% CI = 0.01–0.4, *P* = 0.004; Q4 vs. Q1: OR_adjusted_ = 0.04, 95% CI = 0.01–0.3, *P* = 0.001; *P*_trend_ <0.0001) (Table**** 2B).

We next assessed the association of 25(OH)D_2_ levels with lung cancer status. Multivariable analyses did not indicate any significant associations between 25(OH)D_2_ levels and lung cancer status, although the trends were similar to those observed for 25(OH)D_3_ (*P*_trend_ = 0.08) (Table**** 3A). As previously mentioned, vitamin D_2_ was detectable in only a subset of subjects (139 cases and 156 controls) who might have been exposed to vitamin D_2_ from dietary or supplement sources. Additionally, no significant associations were evident for the 1,25(OH)_2_D_2_ and lung cancer (*P*_trend_ = 0.67) (Table**** 3B).

**Table 3:** Logistic regression analysis of (A) 25(OH)D_2_ and (B) 1,25(OH)_2_D_2_ levels across quartiles

	Univariable				Multivariable			
	*N* (%) cases	*N* (%) controls	OR (95% CI)	*P*‐value^a^	*N* (%) cases	*N* (%) controls	OR (95% CI)^b^	*P*‐value^a^
(A) 25(OH)D_2_								
Q1_referent_	53 (38)	39 (25)	1.0		53 (38)	39 (25)	1.0	
Q2	54 (39)	39 (25)	1.0 (0.6–1.8)	0.95	53 (38)	39 (25)	1.0 (0.6–2.8)	0.61
Q3	25 (18)	41 (26)	**0.5 (0.2–0.9)**	**0.02**	25 (19)	41 (26)	0.9 (0.4–2.2)	0.81
Q4	7 (5)	37 (24)	**0.1 (0.1–0.3)**	**<0.0001**	7 (5)	37 (24)	0.4 (0.1–1.5)	0.18
			***P***_**trend**_ **<0.0001**					*P*_trend_ = 0.08
(B) 1,25(OH)_2_D_2_								
Q1_referent_	22 (43)	12 (25)	1.0		22 (43)	12 (25)	1.0	
Q2	8 (16)	12 (25)	0.4 (0.1–1.1)	0.08	8 (16)	12 (25)	1.0 (0.2–4.7)	0.99
Q3	12 (23)	12 (25)	0.6 (0.2–1.6)	0.27	12 (23)	12 (25)	0.6 (0.1–2.8)	0.52
Q4	9 (18)	12 (25)	0.4 (0.1–1.3)	0.12	9 (18)	11 (25)	0.9 (0.1–5.0)	0.83
			*P*_trend_ = 0.14					*P*_trend_ = 0.67

CI, confidence interval; OR, odds ratio.

a **Statistically significant; ***P***‐value <0.05**.

b Multivariable unconditional logistic regression adjusted for age, gender, race, interview year, smoking status, pack‐years, blood collection month, and vitamin D supplement use.

While this study was not designed to investigate differences in association between vitamin D levels and lung cancer status in African Americans versus European Americans, we nevertheless stratified logistic regression models on race. In African Americans, increasing levels of 25(OH)D_3_ were inversely associated with lung cancer status in the second and third quartiles (Q2 vs. Q1: OR_adjusted_ = 0.4, 95% CI = 0.2–0.9, *P* = 0.03; Q3 vs. Q1: OR_adjusted_ = 0.3, 95% CI = 0.1–0.7, *P* = 0.01; *P*_trend_ = 0.03), while in European Americans, only the highest quartile was inversely associated with lung cancer status (Q4 vs. Q1: OR_adjusted_ = 0.5, 95% CI = 0.2–1.0, *P* = 0.04; P_trend_ = 0.04) (Table S3). No significant observations were seen for associations between 25(OH)D_2_ levels and lung cancer status stratified by race after the adjustment for a number of putative confounders (Table S4). The analysis assessing association between 1,25(OH)_2_D_2/3_ levels and lung cancer status could not be stratified on race because of the small number of subjects, which did not allow for a sufficiently powered analysis.

In order to investigate whether the IOM‐reported reference value of 20 ng/mL total 25(OH)D, levels below which indicate vitamin D deficiency, is associated with lung cancer, logistic regression analysis was performed on categorical variables dichotomized based on 25(OH)D_3_. Levels higher than 20 ng/mL were inversely and significantly associated with lung cancer status in the entire cohort (OR_adjusted_ = 0.5, 95% CI = 0.3–0.8, *P* = 0.005) and in European Americans (OR_adjusted_ = 0.5, 95% CI = 0.3–0.8, *P* = 0.003). While the multivariable results were not significant in African Americans, the effect size and direction indicated a similar trend to that in European Americans (Table S5).

### Associations of SNPs in vitamin D metabolism network genes and lung cancer

**Figure 1:**
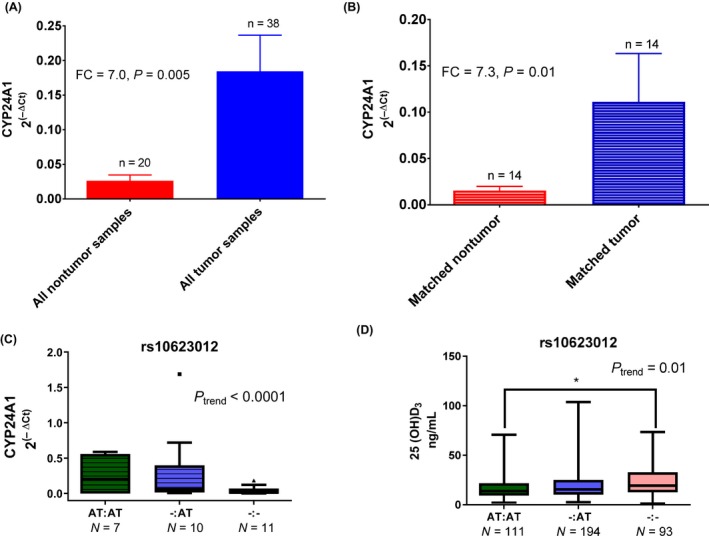
The qRT‐PCR of CYP24A1 in (A) tumor and adjacent nontumor tissue, (B) subset of matched tissue pairs only, and (C) stratified by rs10623012 genotypes. (D) Vitamin D_3_ levels measured by UPLC‐MS/MS stratified by rs10623012 genotypes. *P‐value <0.05.

We genotyped 296 SNPs in CYP2R1, CYP27A1, CYP27B1, CYP24A1, and VDR and observed associations between 27 SNPs and lung cancer status after the adjustment for age, gender, and race, all of which had passed the Hardy–Weinberg equilibrium assessment (Table S6). Eighty‐nine percent of the associated SNPs were located in CYP24A1 and VDR, indicating enrichment of genetic variation in the vitamin D signaling cascade associated with lung cancer in those pivotal genes. Of 27, 13 SNPs demonstrated significant interactions with vitamin D_3_ levels, one of which was rs10623012 in the 3′UTR of CYP24A1 (Table S6). The heterozygous genotype of this SNP was inversely associated with lung cancer status (OR_adjusted_ = 0.7, 95% CI = 0.5–0.9; *P *=* *0.03). The homozygous minor genotype showed a similar but insignificant effect, possibly due to the insufficiently powered analysis. As there is evidence for CYP24A1 functional significance in lung carcinogenesis, we investigated whether mRNA levels of this gene are deregulated in 30 tumor tissue samples when compared to 20 adjacent nontumor tissue samples from the NCI‐MD cohort. We observed a significant overexpression of CYP24A1 in this analysis (FC = 7, *P* = 0.005, Fig. 1A), even when only matched tumor and adjacent nontumor tissue samples were compared (*N* = 14, FC = 7.3, *P* = 0.01, Fig. 1B). Considering that 3′UTRs are of special interest due to the transcriptional regulation of mRNA levels by microRNA interactions with this region, we investigated whether rs10623012 genotypes were associated with CYP24A1 mRNA levels. Indeed, heterozygous and homozygous minor genotypes were associated with lower CYP24A1 mRNA expression (*P* < 0.0001, Fig. 1C). As expected, rs10623012 heterozygous and homozygous minor genotypes exhibited significant upward trend with higher vitamin D_3_ levels, pointing to a possible functional significance of this SNP, and possibly explaining the protective association of rs10623012 in lung cancer (Fig. 1D).

## Discussion

The Institute of Medicine (IOM) reports from 2011 highlighted a significant need for population‐based studies regarding the effect of vitamin D on nonskeletal health outcomes 4. There is an emerging body of evidence suggesting that the hormonally active form 1,25(OH)_2_D plays a significant role in carcinogenesis, by inhibiting proliferation and promoting apoptosis 34, 35, 36. Detailed mechanisms of vitamin D action against carcinogenesis are not yet fully understood. Moreover, the evidence for association between 25(OH)D levels with risk of lung cancer is remarkably inconsistent.

Previously, several studies found no association between circulating total 25(OH)D levels and lung cancer risk 26, 37, 38, while others showed inverse associations 23, 24, 25, 27, 37, 39. These inconsistencies, however, may be largely accounted for by methodological limitations. Namely, the majority of the previous studies investigating associations between vitamin D levels and cancer risk were conducted using radioimmunoassays (RIAs) 16, 37, 40, 41, 42, 43 and chemiluminescence immunoassays (CLIAs) 26, 27, 38, 44, the majority of which measured total vitamin D only. Additionally, many immunoassays suffer from cross‐reactivity between vitamin D_2_ and D_3_ forms, therefore resulting in confounded measurements. Moreover, 24,25(OH)_2_D and other polar metabolites may be included in the total vitamin D measurements 45, thus exhibiting systematic bias and underestimation when compared to LC‐MS/MS 33, 46, 47. While there is little doubt that immunoassays have shown more than 40 years of utility in research and healthcare, it is commonly accepted that vitamin D is a difficult metabolite to measure 48. It is therefore highly likely that mass spectrometry‐based methods with their superior specificity may be the future method of choice in many research and clinical applications of steroid hormone measurements. These limitations were addressed herein by using ultraperformance liquid chromatography‐tandem mass spectrometry (UPLC‐MS/MS) that allowed for separation of vitamin D_2_ and D_3_ forms.

Our goal was to carry out a comprehensive study using sensitive and state‐of‐the‐art method to evaluate whether and what form of vitamin D may play a role in lung cancer. To our knowledge, no study has examined hormonally active 1,25(OH)_2_D levels in relationship to lung cancer, and only a few have been conducted in other cancer types using radioimmunoassays (RIAs) and chemiluminescence immunoassays (CLIAs) 49, 50, 51, 52. Measurements of 1,25(OH)_2_D are of special interest, as it is this metabolite that exhibits tumor‐suppressive properties in cancer. In this study, we found that increasing levels of 25(OH)D_3_ have a significant inverse association with lung cancer status. We also observed that increasing levels of 1,25(OH)_2_D_3_ were significantly and inversely associated with lung cancer status, therefore marking the first report to show a significant association between 1,25(OH)_2_D_3_ levels and lung cancer status. Vitamin D_2_ comes from exogenous plant‐based sources and was therefore measurable in only a subset of subjects who would have been exposed to it from dietary sources or supplements. We, however, did not observe significant associations between 25(OH)D_2_ or 1,25(OH)_2_D_2_ and lung cancer status.

Cognizant of genetic variations in genes in the vitamin D network that may have a direct effect on vitamin D metabolism, we genotyped 296 SNPs in the following genes: CYP2R1, CYP27A1, CYP27B1, CYP24A1, and VDR. Of the 27 observed associations with lung cancer, 89% of the SNPs were located in CYP24A1 and VDR genes, and several of those SNPs were found to significantly modulate vitamin D_3_ levels. This may point to the biological implications of the associated SNPs in affecting vitamin D levels, thereby illuminating the functional significance of their association with lung cancer. CYP24A1 and VDR are key players in the metabolism of vitamin D. Upon exposure to UV light, 7‐dehydrocholesterol is converted to previtamin D_3_ in human skin. In the liver, vitamin D is further metabolized to 25‐hydroxyvitamin D (25(OH)D) by 25‐hydroxylase (CYP27A1). Downstream, 25(OH)D is converted to its active form, 1,25‐dihydroxyvitamin D (1‐25(OH)_2_D) by 1*α*‐hydroxylase (CYP27B1) in the kidneys. Consequently, 1‐25(OH)_2_D increases calcium absorption and exerts effects by interacting with the vitamin D receptor–retinoic acid X receptor complex (VDR‐RXR) to control genes responsible for proliferation, differentiation, apoptosis, and angiogenesis. Additionally, 1‐25(OH)_2_D can also induce the expression of 25‐hydroxyvitamin D‐24‐hydroxylase (CYP24A1), which breaks down both 25(OH)D and 1,25‐(OH)_2_D into biologically inactive calcitroic acid 53.

We were especially interested in genetic variants in CYP24A1 due to its previously observed overexpression in lung and other tumor types and reported association with poorer lung cancer survival 14. A particular SNP came into focus, rs10623012 in the 3′UTR of CYP24A1. As 3′UTR regions are known to be transcriptionally regulated by microRNAs (miRs), we hypothesized that this variant may lead to differences in the mRNA expression. We indeed observed that rs10623012 heterozygous and homozygous minor genotypes were inversely associated with lung cancer status, displayed a significant upward trend with vitamin D_3_ levels and were negatively associated with CYP24A1 mRNA expression. The aforementioned observations may illuminate the functional implication of rs10623012 variant in the context of lung carcinogenesis, namely that the protective associations of this variant with lung cancer may be explained by its modulation of vitamin D_3_ levels. It remains to be investigated whether this SNP leads to a formation of a binding site for any miRs, which would explain its association with lower CYP24A1 mRNA levels.

While the IOM report from 2011 stated evidence suggesting lower levels of total 25(OH)D in African Americans and other darker‐skinned individuals when compared to European Americans, the cut point indicating deficiency was generalized to the entire North American population. It is important to keep in mind, however, that the RDA was based on skeletal health outcomes only. Although our study was not designed to address differences in associations between vitamin D levels and lung cancer status when stratified on race, the data presented herein may provide evidence that cut points for vitamin D levels in the context of lung cancer might need to be established separately in different racial groups. Additionally, we caution that the IOM cutoff of 20 ng/mL indicating vitamin D deficiency should not be extrapolated to other health outcomes.

While this nested case–control study comprises a reasonably large number of cases and controls that were well matched on age and gender, caveats of the study include a low number of African American subjects that prevented stratified analyses on race. Although a number of putative confounders were considered in the present work, residual confounding (e.g., dietary exposures to vitamin D) cannot be ruled out. We, however, controlled for the self‐reported supplement use of vitamin D or vitamin D‐containing multivitamins in all multivariable analyses, as well as for the month of blood collection. The results of this study suggest that an internal mechanism due to vitamin D_3_ tumor‐suppressive properties leads to inverse associations between increasing vitamin D_3_ levels and lung cancer status, rather than supplement use itself. These associations may be directly mediated by genetic variation in VDR and CYP24A1 genes, crucial players in the vitamin D metabolism cascade. The lack of association between lung cancer and vitamin D_2_ is most probably due to differences in the effectiveness of vitamin D_2_ versus D_3_ action at a molecular level: Vitamin D_2_ binds to the Vitamin D Receptor (VDR), a rate‐limiting step in the vitamin D signaling cascade, with a lesser specificity when compared to D_3_ 54. Therefore, it may not be surprising that vitamin D_2_ levels had a less pronounced protective effect in lung cancer. This presents a significant consideration in the future recommendations regarding vitamin D supplementation in relationship to nonskeletal health outcomes and laboratory measurements of specific metabolites of vitamin D, as distinctions between the two forms of vitamin D appear to be relevant.

The novelty of this study includes utilization of a very sensitive and specific method for quantitation and distinctions between the four metabolites of vitamin D: 25(OH)D_2/3_ and 1,25(OH)_2_D_2/3_ by mass spectrometry. Mass spectrometry is becoming a method of choice for vitamin D measurements due to its high sensitivity and specificity, and limited effects by the endogenous and exogenous interferents inherent to many immunoassays. Food and Drug Administration has recently cleared a first mass spectrometry vitamin D assay for clinical use 55. This points to a significant prospect of this technology in clinical practice for measurements of vitamin D, and perhaps other analytes that would benefit from the high sensitivity and specificity of such technology.

We showed that vitamin D_3_ has a stronger association with lung cancer when compared to vitamin D_2_. We also found that vitamin D metabolism may be significantly affected by genetic variation in VDR and CYP24A1 genes. Future studies in additional populations are warranted, especially addressing prospective associations of vitamin D_2_ and D_3_ levels and lung cancer risk.

## Conflict of Interest

The authors disclose no potential conflict of interests.

## Supporting information


**Table S1**. Cohort characteristics of a subset of samples used for quantitation of the active 1,25(OH)_2_D_2/3_ levels.
**Table S2**. Concentration values of inactive 25(OH)D_2/_D_3_, and active 1,25(OH)_2_D_2_/D_3_ in the NCI‐MD case‐control set.
**Table S3**. Logistic regression analysis of inactive 25(OH)D_3_ across quartiles in (A) African‐ and (B) European‐Americans.
**Table S4** Logistic regression analysis of inactive 25(OH)D_2_ across quartiles in (A) African‐ and (B) European‐American subjects.
**Table S5**. Logistic regression analysis of 25(OH)D_3_ dichotomized based on the Institute of Medicine reported reference value of <20 ng/mL of inactive 25(OH)D indicating vitamin D deficiency.

**Table S6**. Logistic regression analysis results of SNPs significantly associated with lung cancer status, and linear regression of SNPs and 25(OH)D_3_ and 1,25(OH)_2_D_3_ levels in the NCI‐MD case‐control cohort.
