# Supplementary material for: Inverse association of vitamin D3 levels with lung cancer mediated by genetic variation
Source: Cancer Med. 2018 May 3;7(6):2764–75. doi: 10.1002/cam4.1444 (PMC6010700; doi:10.1002/cam4.1444)
Supplement: Supplementary file 1 — Table S1. Cohort characteristics of a subset of samples used for quantitation of the active 1,25(OH)2D2/3 levels. Table S2. Concentration values of inactive 25(OH)D2/D3, and active 1,25(OH)2D2/D3 in the NCI‐MD case‐control set. Table S3. Logistic regression analysis of inactive 25(OH)D3 across quartiles in (A) African‐ and (B) European‐Americans. Table S4 Logistic regression analysis of inactive 25(OH)D2 across quartiles in (A) African‐ and (B) European‐American subjects. Table S5. Logistic regression analysis of 25(OH)D3 dichotomized based on the Institute of Medicine reported reference value of <20 ng/mL of inactive 25(OH)D indicating vitamin D deficiency. [file CAM4-7-2764-s001.doc]

**Inverse Association of Vitamin D_3_ Levels with Lung Cancer Mediated by Genetic Variation**

Majda Haznadar^1^, Kristopher W. Krausz^2^, Ezra Margono^1^, Christopher M. Diehl^1^, Elise D. Bowman^1^, Soumen K. Manna^3^, Ana I. Robles^1^, Bríd M. Ryan^1^, Frank J. Gonzalez^2^, Curtis C. Harris^1^

^1^Laboratory of Human Carcinogenesis, Center for Cancer Research, National Cancer Institute, Bethesda, MD, 20892. ^2^Laboratory of Metabolism, Center for Cancer Research, National Cancer Institute, Bethesda, MD, 20892. ^3^Saha Institute of Nuclear Physics, Department of Atomic Energy, Biophysics and Structural Genomics Division, Kolkata, India, 700064.

**Corresponding Author:**

Curtis C. Harris, M.D.

National Cancer Institute

Laboratory of Human Carcinogenesis

Building 37, Room 3068

37 Convent Drive, MSC 4258

Bethesda, MD 20892-4258

Tel: 301-496-2048

Fax: 301-496-0497

Email: [Curtis_Harris@nih.gov](mailto:Curtis_Harris@nih.gov)

**SUPPLEMENTARY INFORMATION**

**Supplementary Table 1.** Cohort characteristics of a subset of samples used for quantitation of the active 1,25(OH)_2_D_2/3_ levels.

| **Characteristics** | **Cases** | **Controls** | **Total** | ***P-*value*^b^*** |
| --- | --- | --- | --- | --- |
| **n**  **Age; mean±se** | 90  65.8 ± 8.9 | 104  67.3 ± 10.1 | 194  66.5 ± 9.5 | 0.30 |
| **Race; n (%)**  African-American  European-American | 46 (44)  58 (56) | 22 (24)  68 (76) | 68 (35)  126 (65) | <0.0001 |
| **Gender; n(%)**  Male  Female  **Smoking Status; n (%)**  Current  Former  Never | 46 (51)  44 (49)  47 (52)  37 (41)  6 (7) | 48 (46)  56 (54)  31 (30)  53 (51)  20 (19) | 94 (49)  100 (52)  37 (19)  90 (46)  67 (35) | 0.06  <0.0001 |
| **Pack years; mean±se**  **Histology; n (%)**  Adenocarcinoma  Squamous cell carcinoma  Non-small cell carcinoma | 43.5±3.8    39 (43)  28 (28)  23 (26) | 13.6±2.4 | 29.0±2.7 | <0.0001 |
| **Stage^a^; n (%)**  I  II  III  IV  Unknown | 25 (28)  13 (15)  9 (10)  9 (10)  34 (37) |  |  |  |

^a^Only cases containing pathology reports were staged. Staging presented in the table is based on the 7^th^ edition of the AJCC staging manual, wherein all cases with sufficient tumor size, metastases status and lymph node involvement data previously staged based on the 6^th^ edition were restaged to the 7^th^ edition for consistency.

^b^Two-sided Χ^2^ test (categorical) or t-test (continuous)

**Supplementary Table 2**. Concentration values of inactive 25(OH)D_2/_D_3_, and active 1,25(OH)_2_D_2_/D_3_ in the NCI-MD case-control set.

**Supplementary Table 3**. Logistic regression analysis of inactive 25(OH)D_3_ across quartiles in A) African- and B) European-Americans.

| **25(OH)D_3_** |  | **Univariable** | | | |  |  |  | **Multivariable** | | |  | |  |
| --- | --- | --- | --- | --- | --- | --- | --- | --- | --- | --- | --- | --- | --- | --- |
| 1. **African-Americans** |  | |  |  |  | | |  | |  |  | |  | |
| Q1_referent_ | 77 (77) | | 74 (38) | 1.0 |  | | | 77 (78) | | 73 (38) | 1.0 | |  | |
| Q2 | 13 (13) | | 51 (26) | **0.2 (0.1-0.5)** | **<0.0001** | | | 12 (12) | | 51 (27) | **0.4 (0.2-0.9)** | | **0.03** | |
| Q3 | 6 (6) | | 49 (26) | **0.1 (0.1-0.3)** | **<0.0001** | | | 6 (6) | | 49 (26) | **0.3 (0.1-0.7)** | | **0.01** | |
| Q4 | 4 (4) | | 19 (10) | **0.2 (0.1-0.6)** | **0.005** | | | 4 (4) | | 18 (9) | 0.8 (0.2-3.6) | | 0.80 | |
|  |  | |  | ***P*_trend_ <0.0001** | | | |  | |  |  | | ***P*_trend =_0.03** | |
| 1. **European-Americans** |  | |  |  |  | | |  | |  |  | |  | |
| Q1_referent_ | 110 (36) | | 36 (15) | 1.0 |  | | | 110 (36) | | 36 (15) | 1.0 | |  | |
| Q2 | 81 (27) | | 53 (22) | **0.5 (0.3-0.8)** | **0.008** | | | 79 (26) | | 53 (22) | 0.6 (0.3-1.1) | | 0.10 | |
| Q3 | 67 (22) | | 64 (26) | **0.3 (0.2-0.6)** | **<0.0001** | | | 67 (22) | | 64 (26) | 0.6 (0.3-1.1) | | 0.13 | |
| Q4 | 48 (16) | | 91 (37) | **0.2 (0.1-0.3)** | **<0.0001** | | | 47 (16) | | 89 (37) | **0.5 (0.2-1.0)** | | **0.04** | |
|  |  | |  | ***P*_trend_ <0.0001** | | | |  | |  |  | | ***P*_trend_ =0.04** | |

^a^Statistically significant; *P*-value <0.05

^b^Multivariable unconditional logistic regression adjusted for age, gender, interview year, smoking status, pack years, blood collection month and vitamin D supplement use.

^c^OR: odds ratio; CI: confidence interval

**Supplementary Table 4. Logistic regression analysis of inactive 25(OH)D_2_ across quartiles in A) African- and B) European-American subjects.**

|  |  | **Univariable** | | | |  |  |  | **Multivariable** | | |  | |  |
| --- | --- | --- | --- | --- | --- | --- | --- | --- | --- | --- | --- | --- | --- | --- |
| 1. **African-Americans** |  | |  |  |  | | |  | |  |  | |  | |
| Q1_referent_ | 10 (44) | | 10 (19) | 1.0 |  | | | 10 (44) | | 10 (19) | 1.0 | |  | |
| Q2 | 6 (26) | | 13 (25) | 0.5 (0.1-1.7) | 0.25 | | | 6 (26) | | 13 (25) | 0.6 (0.1-4.9) | | 0.61 | |
| Q3 | 5 (22) | | 15 (28) | 0.3 (0.1-1.3) | 0.11 | | | 5 (22) | | 15 (28) | 0.9 (0.1-7.4) | | 0.65 | |
| Q4 | 2 (8) | | 15 (28) | **0.1 (0.02-0.7)** | **0.02** | | | 2 (8) | | 15 (28) | 0.1 (0.01-1.8) | | 0.12 | |
|  |  | |  | ***P*_trend_ =0.01** | | | |  | |  |  | | *P*_trend_ =0.26 | |
| 1. **European-Americans** |  | |  |  |  | | |  | |  |  | |  | |
| Q1_referent_ | 43 (37) | | 29 (28) | 1.0 |  | | | 43 (38) | | 29 (28) | 1.0 | |  | |
| Q2 | 48 (42) | | 26 (25) | 1.3 (0.6-2.4) | 0.52 | | | 47 (41) | | 26 (25) | 1.3 (0.5-3.3) | | 0.55 | |
| Q3 | 20 (17) | | 26 (25) | 0.5 (0.3-1.1) | 0.09 | | | 20 (17) | | 26 (25) | 0.9 (0.3-2.4) | | 0.81 | |
| Q4 | 5 (4) | | 22 (22) | **0.2 (0.1-0.5)** | **0.001** | | | 5 (4) | | 22 (22) | 0.5 (0.1-2.2) | | 0.36 | |
|  |  | |  | ***P*_trend_ <0.0001** | | | |  | |  |  | | *P*_trend_ =0.45 | |
|  |  | |  |  |  | | |  | |  |  | |  | |

^a^Statistically significant; *P*-value <0.05

^b^Multivariable unconditional logistic regression adjusted for age, gender, interview year, smoking status, pack years, blood collection month and vitamin D supplement use.

^c^OR: odds ratio; CI: confidence interval

**Supplementary Table 5**. Logistic regression analysis of 25(OH)D_3_ dichotomized based on the Institute of Medicine reported reference value of <20 ng/mL of inactive 25(OH)D indicating vitamin D deficiency.
